# The display effects of patients’ self-assessment on traumatic acute pain on the proportion and timing of analgesics administration in the emergency department

**DOI:** 10.1186/s12245-014-0036-1

**Published:** 2014-09-17

**Authors:** Nik Hisamuddin NA Rahman, Cecilia Ananthanosamy

**Affiliations:** 1 Department of Emergency Medicine, School Of Medical Sciences, USM 16150, Malaysia; 2 Emergency and Trauma Department, Serdang Hospital, Selangor, Malaysia

**Keywords:** Acute pain, Pain score, Analgesic, Triage

## Abstract

**Background:**

Acute pain assessment in the emergency department (ED) is important in particular during the triage process. Early pain assessment and management improve outcome. The objective of this study was to determine the effects of documentation and display of patient's self-assessment of pain using numerical rating scale (NRS) on analgesic use among adult trauma patients in ED.

**Methods:**

A randomized control trial was conducted recruiting 216 trauma patients who presented to ED of two tertiary centers. Pain score was done using NRS for all patients. They were randomized into pain score display group or not displayed in the control. The outcome measured were proportion of patients receiving analgesics and timing from triage to analgesic administration.

**Results:**

The proportion of patients who received analgesics when pain score was displayed was 6.5% more than when pain score was not displayed. This difference was however not statistically significant. However, stratified categorical analysis using chi-square showed that the displayed severe pain group was 1.3 times more likely to receive analgesics compared to the non-displayed group. The mean timing to analgesic administration for the displayed and non-displayed groups were 81.3 ± 41.2 (95% C.I 65.9, 96.7) and 88.7 ± 45.4 (95% C.I 69.0, 108.3), respectively (*p* > 0.05).

**Conclusions:**

The proportion of patients who received analgesics increased when NRS was displayed. However, the pain display has no significant effect on the timing of analgesics.

## 1 Background

The usual scenario in the emergency department (ED) is an overworked doctor, carefully putting history together, examination, and investigations, to arrive at a diagnosis. This is often done with much time constraints and poor attention paid to acute pain management. However, a physician's primary duty is to comfort, manage, and reduce the suffering of patients. This study attempted to address this contradiction, by introducing a simple intervention in the ED. This is an effort to evaluate the effect of displaying pain score, without any other intervention, on analgesic use. As pain score is recommended as a vital sign in many international protocols, it is in a way asking if we can improve acute pain management if we implement the pain score display at triaging in the ED.

## 2 Objectives

The main aim of this study is to determine the effect of documentation and display of patient self-assessment of pain using numerical rating scale (NRS) on analgesic administration. Specifically, we are looking into the effects of numerical pain score display on the proportion of patients receiving analgesics and the timing of analgesic use.

## 3 Methods

This was a cross-sectional randomization study in the ED of two tertiary teaching hospitals with an average annual patient attendance of 140,000. This study was granted ethical approval by the research and ethical committee of the institution.

The following were the inclusion criteria:

(a) Adult ≥ 20 years old

(b) Patients complaining of acute pain secondary to trauma

(c) Patients triaged to non-critical zone only

(d) GCS 15/15

The following were the exclusion criteria:

(a) Patients with language barrier

(b) Patients who were intoxicated

(c) Patients with altered mental status or psychiatric illness

(d) Patient under police custody

(e) Patients with potentially life-threatening diseases or injuries

(f) Non-traumatic causes of acute pain

Patients were carefully selected among whom who have likelihood of presenting with significant acute pain secondary to trauma, while avoiding interference with evaluation and care of patients with potentially life-threatening illness or injury. All patients attending the ED would be triaged by a triage officer at the entrance of the department. Critical patients would be admitted to the resuscitation bay immediately after the triage decision had been made. The semi-critical and non-critical patients would be triaged further including the pain scoring assessment. Acute pain assessment by using NRS is a part of a vital sign monitoring parameter included into the triage protocol of the department. Patients would be given analgesics at the triage counter if they presented with moderate to severe pain. Patients would be up-triaged and admitted to the treatment cubicle if they developed worsening of pain while waiting to be seen by doctors. Only patients who were able to give consent and had a clear mind to understand and do pain assessment were included in the study. Randomization was done prior to selection of patients. Patients were assigned to display pain score and non-display pain score groups, using a computer-generated randomization method, in blocks of four. These randomized selections were sealed in opaque brown, non-transparent, numbered envelop and arranged in ascending order. The envelopes were opened only after pain assessment was done and documented by researcher. The researcher and the triage officer were blinded to the randomization while doing the pain score and initial data collection. This was important to avoid bias when pain score was being performed. Verbal consent was obtained from each patient.

The sample size was calculated by using the following parameters:

(1) Po=0.5

(2) Pi=0.3

where *α* = 0.05 (95% CI), study power = 0.8 (80% ), two proportion samples Po = 0.5 and Pi = 0.3, and *M* = 1.

The sample size required were 220 patients (110 per arm) including the 10% dropout.

### 3.1 Data collection

**Figure 1:**
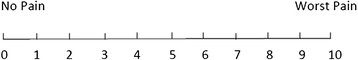
Numerical rating scale (NRS).

The NRS was used as the pain scale assessment tool as this has been assessed as the best tool to be used among trauma patients in the ED. (Figure 1) [1]. A bold red marker pen was used to display the pain score. A yellow sticker measuring 10 × 7 cm was used to attach the pain score at the top right hand corner of the front cover of medical case notes. This pain score was displayed prominently in the patients' medical clerk sheet in one group and not displayed in another group.

Pain assessment was done at triage after chief complaint and vital signs were assessed. This was done by the patients, without relying on physicians' impression. This is as suggested by the Canadian Association of Emergency Physicians (CAEP) consensus document on emergency pain management [2].

Instructions on how to do a pain score was explained to the patients by the researcher. Instruction was given by using local languages and dialects. Relatives were discouraged from translating or trying to answer for the patient. The numerical rating scale was shown to the patient. Patients were asked to give pain score value according to their own assessment of pain severity. They were excluded from the study if they did not understand the instructions after being repeated twice. The data was entered into the data collection form. Based on the previous study carried out by Silka et al., we decided that the NRS score of 7 to 10 is severe pain, NRS score of 4 to 6 is clinically significant moderate pain, and any score of 3 and below is mild pain.

Data entry and analysis were done using Statistical Packages for Social Science (SPSS) version 12.0.1 software which was registered by the institution. Numerical pain score was converted to categorical variable. Chi-square test was used in the analysis of the differences of analgesic administration between the displayed pain score and not displayed pain score groups. The confounding effect of pain severity on this relationship was analyzed by using Mantel-Hanszel estimate. Timing to analgesics was presented as mean and analyzed by using ANOVA test. Statistical significance was considered at *p* value of less than 0.05. Data exploration was done using descriptive statistics and presented as charts and tables for each variable.

## 4 Results

**Figure 2:**
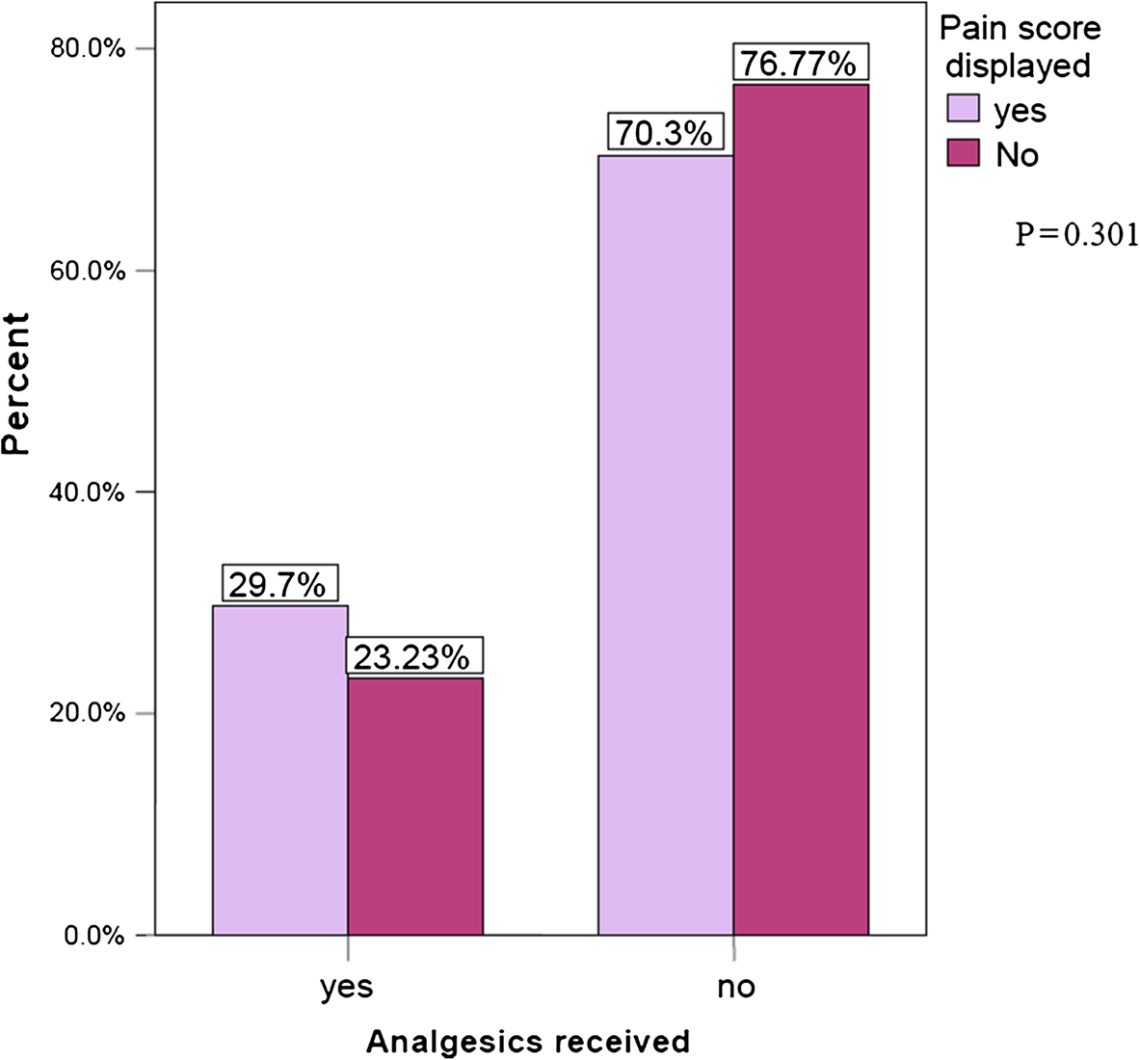
Bar chart showing percentage of patients who received analgesics in trial versus control group.

The number of patients who met all criteria was 216. Finally, there were 107 patients (50.5%) in the study group and 99 (49.5%) patients in the control group after exclusion due to various reasons. The general demographic data comparison between the two study groups is as shown in Table 1. There is no significant difference in the gender distribution, age group, education, mechanism of injury, and pain score between the study groups (*p* > 0.05). Majority of patients who presented with acute pain in this study group were in the range of 20 to 29 years of age (*n* = 99, 49.5%). The distribution of the pain score groupings in the displayed NRS group for the mild, moderate, and severe pain are 12.9% (*n* = 13), 46.5% (*n* = 47), and 40.6% (*n* = 41), respectively. Likewise, the distribution of the pain score groupings in the non-displayed NRS group for the mild, moderate, and severe pain are 17.2% (*n* = 17), 49.5% (*n* = 49), and 33.3% (*n* = 33), respectively. The proportion of patients who received parenteral analgesics for mild, moderate, and severe pain in displayed group was 7.7%, 21.7%, and 47.6%, respectively (*p* < 0.05). Likewise, there is an increment of proportion who received analgesics in the non-displayed group from 23.5% to 31.3% with increasing pain severity (*p* < 0.05). The proportion of patients who received analgesics when pain score was displayed was 6.5% more than when pain score was not displayed. This difference was however not statistically significant (*p* = 0.30) (Figure 2 and Table 2).

**Table 1:** Demographic comparison between the study and control groups

**Variables**	**Non-display (control)group (*n*** **= 99)**	**Display (trial) group(*n*** **= 107)**	***p*** **value**
Age in years (mean)	35.6	38.6	0.639
Gender distribution (*n* = Male or Female)	Male = 70 (70.1%)	Male = 73 (68.2%)	0.242
	Female = 29 (29.2%)	Female = 34 (31.8%)	
Education at minimum secondary school level	59 (59.6%)	64 (59.8%)	0.662
Mean (s.d) pain score (NRS)	5.7 (1.9)	6.1 (2.6)	0.300
Severe pain score (more than 7) (*n*)	33 (33.3%)	41 (38.3%)	0.492
Motor vehicle crash patients (*n*)	51 (51.5%)	42 (39.3%)	0.621
Patients received intravenous analgesics (*n*)	49 (49.5%)	58 (54.2%)	0.310

**Table 2:** Odd ratio of patients receiving analgesics

	**Crude odd ratio**	**Weighted odd ratio**	***p*** **value**
Displayed pain score × Receiving analgesics	1.396		0.30
Pain group^a^ displayed pain score × Receiving analgesics		1.306	0.42

^a^Confounder effect of variable (confounder effect of pain severity on displayed pain score was analyzed using Mantel-Hanszel estimate).

The mean timing (in minutes) to analgesic administration for the displayed and non-displayed groups were 81.3 ± 41.2 (95% C.I 65.9, 96.7) and 88.7 ± 45.4 (95% C.I 69.0, 108.3), respectively (*p* = 0.538). The distribution of timing of analgesics according to the severity of pain is as shown in Table 3.

**Table 3:** Test results of analysis of timing to analgesics by displayed stratified Pain Score using ANOVA

**Display pain score**	**Mean (minutes)**	**Std. deviation**	**95%CI (upper, lower)**	**Minimum**	**Maximum**
Pain score 4 to 6 (displayed)	84.2	51.8	16.4, 47.2	20.0	190.0
Pain score 4 to 6 (not displayed)	89.6	40.4	55.8, 123.4	25.0	170.0
Pain score > 6 (displayed)	80.8	36.0	8.0, 63.9	20.0	165.0
Pain score > 6 (not displayed)	94.3	48.6	72.7, 96.3	25.0	174.0

*p* = 0.946.

## 5 Discussion

Painful suffering is neither a diagnosis nor a disease. Nevertheless, it is a condition experienced by patients posttrauma that needs to be attended to attentively. Over the last few years, there has been much progress in the understanding of the pathophysiology of pain and its effect on disease and healing. More importantly, evidence has emerged on the detrimental effect of neglecting pain on disease progression and patient recovery [3],[4]. There have been many attempts to introduce ways to improve pain management in the ED. Great steps to improving pain and suffering in a patient start with understanding what pain means to the patient. Pain is what the patient states it is. Physicians must respect this. A diverse spectrum of psychological, sociocultural, temporal, and situational variables affects how people perceive and express their pain [5],[6]. The patients' self-report is the most reliable indicator of the presence and intensity of pain. Health care professionals often fail to routinely assess and document pain. Physicians should trust the patients' subjective reports of pain unless there is evidence to the contrary [7],[8].

Few studies had shown the positive effects of pain score display on analgesic use in the ED. In a retrospective study by Nelson, the effect of introducing a mandated verbal numeric pain scale on the incidence and timing of analgesic administration in trauma and non-trauma patients in the ED was studied. Analgesic use increased from 25% to 36%, and analgesics were administered more rapidly after the scale was introduced [9],[10]. Considering the positive results shown by these studies, it was felt that a randomized control study to determine the effect of documentation and display of patient self-assessment of pain using NRS, on analgesic administration was an important move to increase the evidence of the importance of pain score as a fifth vital sign in triage. In this study, a detailed analysis of the three different pain score groups, minimal, moderate, and severe pain, has shown that with the increasing pain score, more patients received analgesics. This is more frequent in the displayed pain score group. In Silka's study in 2004, no patient who reported mild pain received analgesics, whereas 72% of patients with moderate to severe pain did receive analgesics. A similar study was done in 2004 by Thomas. That study used the visual analog scale (VAS), which was displayed at the patient's ED chart and placed at the head of the ED stretcher. The study by Thomas showed that 63% of patients in the group with graphical display of VAS score received analgesics compared to 58.7% in tabulation group and 55.7% in the control group [11],[12]. Our study has found that analgesic administration is significantly associated with increasing severity of pain. This study gives the benefit of knowing what the pain score was in all patients. In patients where pain score was not displayed, a significant proportion of patients with moderate and severe pain still received analgesics. This shows that there are other factors influencing the attending medical officer to prescribe analgesics. Clinical judgment, the doctors' own assessment of pain, seems to have played a significant role here. What can be concluded here is that more patients received analgesics with increasing severity of pain, whether the pain score was displayed or not. It is not only the awareness of a displayed pain score that increased the likelihood of analgesics but also the clinical judgment of the presence of pain is an important factor.

However, in this study, the displayed pain score did not affect the timing to analgesics compared to when pain score was not displayed. The study by Silka also showed no difference in the timing to first analgesics when comparing those patients with or without VPS. Pain assessment using NRS at the time of triage and prominent display of the pain score in patient medical record did not alter the timeliness of analgesic administration. Time to analgesics has not been studied in the ED in this country previously as far as the author's awareness. There is no nationwide system currently in place to triage a patient with a high pain score to see the doctor earlier. There are also no guidelines to administer analgesics earlier in patients with high pain score, while they wait for doctors. These are the measures that could be introduced to reduce the time the patient is in pain while waiting for analgesic administration.

### 5.1 Limitations

The main limitation of this study is the practice of analgesic administration in the ED being influenced by the awareness that the study is going on. The presence of the researcher in the triage and the new sticky label on the medical case notes with the pain score developed the awareness that they were being monitored. Perhaps this created some pressure to give more analgesia than usual or otherwise. This inevitable Hawthorne effect was counterbalanced by the utilization of a control group. Notably, patients and physician were blinded to the study null hypothesis. Only patient factors that influenced pain score and analgesic administration were studied. Factors regarding the doctors' experience or knowledge of pain management were not analyzed. These factors could not be studied as there was a high turnover of doctors in the ED and many different doctors work for the three shifts. Other factors that may influence analgesic administration include the number of doctors on duty and the number of patients on the days of study or particular shift. If there were many patients and the doctors were very busy, they might ignore the pain score and try to work fast in their limited time per patient. The time of the day when the study was conducted may also affect the outcome. These were not considered in the study.

## 6 Conclusions

This study has determined the effect of documentation and display of patient self-assessment of pain using NRS on analgesic administration. The proportion of patients who received analgesics increased when NRS was displayed. However, the pain display has no significant effect on the timing of analgesics.

## Competing interests

The authors declare that they have no competing interests.

## Authors’ contributions

NHR involved in the conception of the study and preparing the manuscript. CA involved in data collection and statistical analysis. Both authors read and approved the final manuscript.
